# The impact of having natural teeth on the QoL of frail dentulous older people. A qualitative study

**DOI:** 10.1186/1471-2458-12-839

**Published:** 2012-10-02

**Authors:** Dominique Niesten, Krista van Mourik, Wil van der Sanden

**Affiliations:** 1 Department of Global Oral Health, College of Dental Sciences, Radboud University Nijmegen Medical Centre, PO Box 9101HB, Nijmegen, The Netherlands; 2 Department of Public Health and Primary Care, Leiden University Medical Centre, Leiden, The Netherlands

**Keywords:** Aged, Oral health, Frailty, Quality of life, Natural teeth, Body image, Self-worth

## Abstract

**Background:**

In order to adapt oral care and treatment to the demands of the growing group of frail dentulous older people, it is important to understand how and to which extent having natural teeth contributes to the quality of life (QoL) of frail older people and how frailty influences their perspective.

**Methods:**

A qualitative approach was used. Interviews with 38 Dutch frail older dentulous people were tape-recorded, transcribed, coded for content and analyzed. Additional information was collected which included age, gender, living situation, use of dental prostheses, self-reported oral health status, chronic disorders, and an index for frailty.

**Results:**

Seven themes were identified in the relationship between natural teeth and the QoL of the participants: pride and achievement; intactness; sense of control; oral function; appearance; comfort; along with coping and adapting to disabilities. Having natural teeth generally had a positive effect on QoL. Positive effects through pride and achievement, intactness, and sense of control were most apparent for the most severely frail. They compared themselves with peers who are more often edentate, and valued the good state of their teeth against the background of their declining health, especially those with disabilities causing severe chronic pain or impaired fine-motor skills. The effect of coping with and adaptation to tooth loss was also most apparent for the most severely frail. There was a gender effect in that the men generally cared less about having natural teeth than women, regardless of their level of frailty.

**Conclusions:**

QoL of frail older people is positively influenced by natural teeth, and this effect seems to increase with increasing frailty. Preservation of teeth contributes to a positive body image and self-worth. Oral care for frail people should aim to preserve natural teeth if possible.

## Background

The type and level of oral health care that is currently provided for the fast growing group of frail dentulous older people is not tailored to their treatment needs and demands [1,2]. Since more older people retain their natural teeth, the objective need for dental treatment for this group increases. This applies in particular to frail people, since medication use, systemic diseases and a weakened physical and cognitive condition make frail people more vulnerable to the impact of oral disorders [3,4]. Frailty, being “a state of reduced psychological or physical reserve in combination with an increased risk for adverse outcomes such as falls, disability, and institutionalization” [5] is likely to change the experience of health in general [6-8]. Likewise, frailty is expected to change the value that people ascribe to their oral health and to having natural teeth, and will consequently influence subjective dental care needs and demands.

Associations found between frailty and oral health related QoL have been studied [9-11] but mostly with quantitative surveys where standard ‘oral health related QoL’ (OHrQoL) instruments, such as the Oral Health Impact Profile (OHIP) [12] or the Geriatric/General Oral Health Assessment Index (GOHAI) [13]. These instruments focus almost exclusively on the negative impacts of oral disease [14,15] and thus fail to assess the positive contribution that natural teeth can make to QoL. Another limitation of the instruments is that they do not identify positive or neutral attitudes to oral health despite negative oral health impacts. For instance, not all people avoid social situations when they miss a front tooth. This may be due to changed expectations of health in old age [16], coping and adaptation skills [17,18], or a generational effect in people who experienced much hardship during their formative years, especially among the “war generation” who may be more resilient to change than younger people [19]. These positive attitudes could provide an explanation for the discrepancy between self-rated oral health status and OHrQoL measures [16,19,20].

Coping, adaption and expectancy generally have a stronger effect with increasing age and frailty to influence the personal and dynamic nature of QoL [18]. The WHO has defined QoL as “*an individuals*' *perception of their position in life in the context of the culture and value systems in which they live and in relation to their goals*, *expectations*, *standards and concerns*” [21]. Locker [22], Bowling [23] and Browne [24] have argued that QoL has only meaning at a personal level, and that related domains of significance should be determined individually for everyone to gain deeper insights to the impacts of specific health aspects (like having natural teeth) on QoL.

The relationship between natural teeth and QoL has been addressed in several enquiries conducted either with psychometric instruments [9,20,25-27], or by personal open-ended interviews [28,29]. Most participants in MacEntee’s study [28] indicated that they wished to maintain their natural teeth as long as they did not cause problems. This finding was supported by surveys elsewhere showing that higher numbers of natural teeth [9,20,26,27], and higher numbers of occluding pairs of natural teeth [30] are associated with better OHrQoL scores. Likewise, studies on tooth loss [31-33] showed that loss of teeth often negatively influences QoL of older people, e.g. through impaired eating function, lowered self-confidence, and dislike of appearance. However, it is not yet clear *how* having natural teeth contributes to the QoL of frail older people, nor has the influence of frailty in this relationship been assessed. This knowledge, together with other relevant information, will help to identify frail elderly who are likely to benefit most, in terms of QoL, from oral care support or treatment, and thus allocate resources more efficiently.

Consequently, we posed the following research questions: “How do natural teeth contribute to the QoL of dentulous people who are elderly and frail” and “How does frailty influence the impact of having natural teeth on QoL”.

## Methods

Since our research questions target the experiences and perspectives of frail older people with regard to having natural teeth and QoL, a qualitative approach through open-ended interviews was appropriate [34,35]. We used a purposive sampling strategy in order to optimize diversity in responses to our research questions [36]. Hence we selected individual men and women of different ages, cultural background, and different levels of frailty [9,37,38]. Two trained interviewers (DN, KM) conducted open ended interviews. They made ‘field-notes’ immediately after each interview to record their personal reflections on the interview and on extraneous events that might have influenced the interview.

### Setting and participants

DN initially contacted care managers at randomly selected daycare centers and assisted-living homes in East-Netherlands. Most contacted managers consented to cooperate. Participants were then recruited by the care managers, whom we consulted to identify potential recruits from the type and intensity of care they receive, based on the indication of the Dutch National Centre for Indication of Care Need (CIZ). Each resident has a ZZP score determined by a medical authority whereby “1” indicates mild frailty and “6” severe frailty (Table 1). People with ZZP 7–10 were excluded from our interviews because their cognitive status precluded the possibility of an interview or their physical status was beyond ‘frail’. We included residents who were 65 years and older, had at least four natural teeth, were cognitively alert and consented in writing to participate. According to the care managers, almost all of the recruits consented to participate; reasons for non-participation were not communicated. All of the participants before they were asked to sign the Consent Form were informed about the purpose of our study and the methods we would use to interview them and analyze the results as approved by the Medical Ethics Committee (CMO) of the UMC Nijmegen (CMO ref. 2009/153).

**Table 1:** Type and Intensity of Care per ZZP category

**ZZP**	**Assistance**		**Care**			**Medical care**	**Behavioural disorders**	**Care indication Hours/wk**
	**Social coping**	**Psychosocial functioning**	**Personal care**	**Mobility**	**Motoric functioning**			
1	+	0	+	+	0	0	0	3-5
2	+++	+	++	+	+	+	0	5,5-7,5
3	++++	++	++++	+++	++	+	0	9,5-11,5
4	++++	+++	++	+	+	+	+	11-13,5
5	+++++	++++	++++	++++	++	+	+	16,5-20
6	++++	+++	+++++	+++++	+++	++	0	16,5-20

“0” means that no care is needed in the referred category. “++” = coaching needed; “++++” = support needed; “++++++” = staff taking over.

Source: Zorgzwaartepakketten V&V Enschede 2010 PJ/10/1657/imz.

### Interviews

Depending on the wish of the participant, interviews took place at a separate room in the day care centre or assisted living homes, or at people’s own rooms or homes. Confidentiality and anonymity were guaranteed at the start of the interview. Interviews were audio-taped, transcribed verbatim and anonymized. An interview guide was used by the interviewer to prompt questions about: (i) self-reported oral and general health; (ii) the meaning of QoL; and (iii) the significance of natural teeth. Participants were encouraged to give as much information as possible in response to these issues and raise any further related topic. Additional data were collected on each participant’s age, gender, chronic disorders, use of dental prostheses, and ZZP scores.

### Data analysis

The data were analyzed using thematic analysis: The interviewers analyzed the verbatim transcripts of interviews to identify specific themes and the context in which the themes influenced the participant’s QoL and feelings about natural teeth [36]. The interviewers independently coded each transcript line-by-line, before discussing and reviewing the attributes and meaning of the codes until consensus was reached. The coding frame developed throughout the process of data analysis. We used a computer-software program (MaxQDA 2007; http://www.MaxQDA.com) to help keep track of the coding and to enable (semi-) quantification during the analysis. A third investigator (WS) checked the reliability of the codes on a random selection of 5 interviews. Finally, we grouped codes into conceptual themes, which were iteratively checked against the data, refined, and discussed among all authors until we agreed about a final set of themes.

Quotes that best illustrated these themes, or points of distinction within themes, were translated into English, and are included in the ‘results’ section below.

We examined the contribution of having natural teeth to QoL in two ways: by directly asking participants what they felt about natural teeth and QoL; and by identifying, analyzing and comparing segments of text that explicitly or implicitly addressed the value of having natural teeth. Likewise, we assessed how frailty influenced the relation between having natural teeth and QoL in two ways: by comparing the transcripts of participants with different levels of frailty; and by identifying, analyzing and comparing segments of text that explicitly or implicitly addressed the role of frailty. In this context we distinguished between slight frailty (ZZP “1”), moderate frailty (ZZP “2” and “3”), and severe frailty (ZZP “4”, “5”, and “6”).

### Trustworthiness and reliability

We used several triangulation methods to ensure the trustworthiness and reliability of our analysis [39]. Investigator triangulation was achieved through having three researchers analyze the data and discuss interpretations. Within-method triangulation was achieved through combining the findings of observational notes, interviews, and, occasionally, short feed-back sessions with contact persons. Reliability was further enhanced through the consistent use of techniques such as paraphrasing and summarization for clarification during the interviews [36] and by increasing the credibility of interpretations through the use of participants’ quotes and in-vivo codes and (sub) themes [40].

### Reflection on the role of researchers

The research group comprised a multidisciplinary team with extensive experience in and knowledge of qualitative methodology, health sociology and medical anthropology, philosophy, and with both academic and (dental) clinical expertise. Data analysis was thus influenced by knowledge and experience from varying academic and professional backgrounds. Consultation of geriatric dentists and geriatric nurses during the study set up helped us to raise appropriate issues during the interviews, and to better understand the context of responses. The only dental professional of the team did not conduct the interviews.

## Results

### General

Participants were interviewed between March 2009 and August 2010. We stopped after 38 interviews when it was obvious that no new themes were emerging from our analyses (theme saturation) [34]. Apart from two women of Indonesian heritage (born Indonesians who moved to the Netherlands around their thirties), all of the 27 women and 11 men interviewed were of European heritage, and they lived either at home and frequented a day care centre (n=18) or in assisted living homes (n=20). They had an average age of 79.9 years (65 – 97 yr), with varying degrees of frailty and a wide range of chronic disorders (Table 2). Most of them had removable partial dentures or complete upper dentures. Information on fixed dentures was not available for all participants and is therefore not included. Table 2 shows an overview of characteristics of all participants.

**Table 2:** Characteristics of participants

	**Gender**	**Age**	Ethnic group*	**Cardiovascular diseases**	**Arthritis/ rheuma**	**Diabetes**	**Cancer**	COPD**	**Psychological dysfunction**	**Parkinson**	**Paralytic**	**Other (e.g auditive or visual impairment)**	**Impaired fine motor skills**	**Impaired gross motor skills**	**ZZP (frailty index)**	**Home/Institute**	**Dental status**
1	**f**	**69**	**C**		x									x	1	H	RPD
2	**m**	**69**	**C**				x								1	H	N
3	**f**	**76**	**M**	x		x									1	H	N
4	**f**	**80**	**C**											x	1	H	CUD
5	**f**	**75**	**C**	x											1	H	RPD
6	**m**	**84**	**C**									x			1	H	C+R
7	**f**	**80**	**C**	x										x	1	H	N
8	**m**	**72**	**C**	x	x	x									1	H	RPD
9	**f**	**86**	**C**		x	x									1	I	N
10	**m**	**72**	**C**						x						1	H	RPD
11	**m**	**69**	**C**				x								1	H	N
12	**m**	**88**	**C**		x									x	2	H	N
13	**f**	**78**	**C**						x						2	H	CUD
14	**f**	**85**	**C**	x			x								2	H	C+R
15	**m**	**79**	**C**	x								x		x	2	H	C+R
16	**m**	**67**	**C**						x						2	I	C+R
17	**f**	**84**	**C**						x					x	2	I	N
18	**f**	**79**	**C**						x					x	2	I	CUD
19	**f**	**94**	**C**									x		x	2	I	CUD
20	**f**	**84**	**C**	x	x							x		x	2	I	C+R
21	**f**	**72**	**C**								x	x		x	3	H	RPD
22	**f**	**83**	**C**					x						x	3	I	CUD
23	**m**	**78**	**C**		x						x	x	x	x	3	H	RPD
24	**f**	**69**	**C**		x							x		x	3	H	N
25	**m**	**80**	**C**							x			x	x	4	H	RPD
26	**f**	**90**	**C**									x		x	4	I	RPD
27	**f**	**97**	**C**		x						x			x	4	I	N
28	**f**	**93**	**C**	x				x				x	x	x	4	I	RPD
29	**f**	**93**	**C**						x		x			x	5	I	N
30	**f**	**84**	**C**	x					x			x			5	I	RPD
31	**f**	**79**	**M**						x					x	5	I	N
32	**f**	**85**	**C**		x						x			x	6	I	CUD
33	**f**	**70**	**C**							x			x	x	6	I	N
34	**f**	**83**	**C**						x					x	6	I	N
35	**f**	**82**	**C**						x					x	6	I	RPD
36	**f**	**88**	**C**						x		x			x	6	I	N
37	**f**	**77**	**C**		x								x	x	6	I	N
38	**m**	**65**	**C**		x				x		x	x	x	x	6	I	CUD

*C= Caucausian, M = Mongoloid; **Chronic Obstructive Pulmonary Diseases.

Dental status: RPD= removable partial denture(s), CUD = complete upper denture, C+R = complete upper denture and removable partial denture(s), N = natural teeth only.

### Quality of life

When asked about what constituted QoL for them, participants’ initial replies varied from “*seeing my grandchildren twice a week*” and “*reading books*” to more general factors like “*being independent*” and “*good health*”. Participants’ answers could be roughly divided into the domains physical health, psychological well-being, social participation, autonomy, and being active. Most participants mentioned at least 2 or 3 domains or related items. Health, autonomy and social participation were most frequently mentioned. Typically, the least frail put more emphasis on the importance of being healthy and less on participation; while generally it was the other way around for severely frail people:

“I like the fact that I can still walk up and down the alley with the rollator walker. That I don’t need help when I go to the restaurant. That I can go out of my room and see people and do crosswords with a friend, that is very important to me.” (woman, 97, severely frail)."

The realization that good health is unattainable for most severely frail people often moderated their ‘priority list’ relating to QoL.

“Good health, that is the most important good, but I will never have that anymore. Yet, I can still join all the parties that take place here. And I do get a lot of pleasure from that. Any time that something is going on, I join in. I do the conga in my wheelchair.” (woman, 77, severely frail)."

When asked how having natural teeth contributed to what the participant thought a good QoL entailed, a variety of answers followed. Before analyzing these answers in detail, it is important to note that basically all participants saw oral health as a part of their general health, and only made a gradual rather than a principal difference between one and the other where impact on their QoL was concerned:

“A healthy mouth is very important to me. That has to do with the overall condition of my body. The mouth is part of a whole.” (man, 69, slightly frail)."

### Value of having natural teeth

It appeared that having natural teeth generally contributed to people’s QoL in a positive sense. We identified six themes that addressed the relationship between having natural teeth and QoL: achievement and pride; sense of control; intactness; oral function; appearance and comfort. Furthermore, the mediating effects of adaptation and coping to experienced tooth decline or loss, and of acceptance of anticipated tooth decline or loss, emerged as a separate theme.

#### Achievement and pride

Having preserved natural teeth gave people a sense of achievement which inspired pride:

“Yes I do feel proud that I have been showing discipline in looking after my teeth, that I have always done my best to look after my teeth as well as I could. Many people are indifferent, careless, because it requires an effort, it is a hassle to look after your teeth. And I have overcome that aversion.” (man, 72, slightly frail)."

Many participants across all frailty categories mentioned this sense of achievement and, in many cases, with pride by comparing themselves to peers who did not have their natural teeth. There was an assumption that people without natural teeth had not put in the same effort to preserve their teeth. This comparison with edentulous people seemed to generate even more pride for people with impaired motor skills:

“I do make the effort to brush [my teeth] every night, even though my hands give me awful pain, I suppose that is very brave of me.” (woman, 70, severely frail, severe Parkinson)."

There was pride also in being exceptional compared to others of the same age or level of frailty by having natural teeth:

“I am quite proud to still have my own teeth, because everyone thinks I have dentures. And almost everybody does indeed have dentures here […]. Every nurse asked me where I left my dentures at night. I said: ‘I have no dentures.’ They didn’t believe it. It is very exceptional, it really is.” (woman, 77, severely frail, institutionalized)."

This pride was expressed typically by participants who were severely frail and institutionalized, who compared themselves downwardly with others:

"“I enjoy having preserved my teeth […] because I have noticed that most people of my age have dentures, and even quite a few people who are much younger than I am.” (woman, 84, severely frail, institutionalized)."

Most people said that retention of natural teeth was not only a matter of achievement but also a consequence of environmental factors and genes (several people mentioned that toothbrushes, toothpaste and particularly fluoridated toothpaste, had not been available to them until their late youth or early adulthood, due to the War and lack of money). Participants who mentioned the influence of environmental factors and genes, still felt that “good teeth” were an achievement due primarily to persistent and good oral care, and the awareness of this achievement, like the awareness of being exceptional, contributed to their sense of self-worth (“a reflection of a person's overall evaluation of his or her own worth” [41]) which was evidenced through comments like “*the fact that I still have my natural teeth does make me feel better about myself*.” (woman, 97, severely frail, partially paralyzed, institutionalized).

### Sense of control

A “*sense of control*” for the participants meant being responsible for maintaining good teeth.

“I’m happy that I can brush my own teeth. You do it for yourself, after all. You need to look after what you’ve got. That goes for the whole body. It is satisfying.” (woman, 94, moderately frail)."

They wanted to look after their teeth and linked their sense of control to their autonomy and independence, which were mentioned frequently as important contributions to QoL.

We identified two reactions to the *thought* of losing control: Acceptance of help for oral hygiene to preserve natural teeth, and a preference for dentures rather than being dependent on others to maintain natural teeth. The former was the dominant reaction, generally from women and others who were quite frail. Yet there was a difference between the thought of losing control and the experience of losing it. One younger woman with severe Parkinson disease, who had problems coping with and accepting her disease, indicated how losing control made her anxious:

“I want to keep doing everything myself, combing my hair, cutting my nails, brushing my teeth. Very often, I can’t do it. And then I get very angry, very angry, even though I know that the nurses who do it for me can’t help it […]. It’s not good, I know.” (woman, 70, severe Parkinson, severely frail)."

A few people who suffered from physical pain caused by chronic complaints stressed the importance of maintaining some control over their teeth since they constituted a part of the body that they still controlled:

“I find it very important to maintain control over my own teeth. If you look at all that I had to give up…so much. But I am still the boss over some parts of my own body. When I don’t think it’s a good idea to eat a sweet, I won’t take it.” (woman, 77, severe arthritis, severely frail)."

Although the thought of losing control was accepted generally with less difficulty by severely frail participants, the idea of maintaining their natural teeth was particularly important against the backdrop of declining health. Control was related to oral health in general but also as something of value for itself. There was a more subtle feeling of responsibility for keeping *intact* body parts (such as teeth) healthy:

“I want to look after my teeth. They belong to me. And anything that belongs to me, I wish to take care for.” (woman, 84, moderately frail)."

Assessment of natural teeth was not always purely positive because a few participants believed that it was easier to maintain dentures. However, the satisfaction of being able to maintain self-control far outweighed the inconvenience that it entailed.

#### Intactness

Numerous participants across all frailty categories mentioned that they felt good or wholesome when teeth were still intact, or incomplete when teeth were missing:

“It [Missing my teeth] is like something is lacking; it is not all complete anymore, isn’t it. […] That is a pity, I really do regret it, even though it doesn’t cause me real trouble.” (man, 88, moderately frail, 3 molars missing)."

“I used to have good teeth, but I did not have the opportunity and the money to have them restored, which I regret. The better your body is preserved, the better you feel.” (woman, 85, moderately frail, full upper dentures)."

Several participants were upset to have lost natural teeth, even if the loss did not cause functional problems or if they had dentures, because they felt incomplete. A few, mostly male or severely frail, participants said that tooth-loss did not bother them. But, overall, the term “false teeth” was considered pejorative, and the idea of removing them from the mouth somewhat revolting:

“*I find it repulsive*, *when people take out their dentures*. *Or rinse them under the tap*, *brrr*.” (woman, 76, slightly frail).

Natural teeth, in contrast, bestowed a sense of dignity “*I think that it* [keeping your natural teeth] *is part of being human*.” Having preserved one’s teeth gained importance against the background of a declining body for a number of severely frail people, especially for those with chronic pain and those who had problems accepting their poor health:

“Having your own teeth, that means: a bit of self-preservation, you feel better about yourself. It means preservation of that small part of your body, while the rest is collapsing.” (woman, 70, severe Parkinson, severely frail)."

It was better, we heard, to have natural teeth because “*what is body*-*own is best*”, and “*natural teeth always fit*, *because they belong to you*, *like your arms and legs*.”

Having natural teeth thus contributed to a more positive body image through feelings of bodily integrity and wholeness. Losing teeth, on the other hand, negatively changed perceptions pertaining to body image for several participants.

#### A mouth that functions

Oral function is an important domain within all oral health related QoL instruments and it is not surprising that basically everybody in this study mentioned the importance of a mouth that functions in relation to QoL. The contribution of natural teeth to oral function was often determined in contrast with dentures. Many thought that good function is related to teeth being well fixed and fitted, and that dentures do not “fit” like natural teeth:

“If I would have full dentures, I expect that would be annoying, because of all the discomfort , that they would not fit well, that I wouldn’t be able to chew, or eat properly, that they would be a bit loose, such things.” (man, 84, slightly frail)."

The function most mentioned was eating, followed by talking. Smiling, kissing and laughing were also closely associated with QoL. One paraplegic man revealed some situational benefits of natural teeth:

“I do a lot with my mouth, like carrying things. When I need a milk carton from the fridge, I grab it with my teeth. If I would take it with my hands, it could easily fall […]. I have also seen other people in the rehabilitation clinic who cannot use their hands at all. They do a lot with their mouth, the ones that have strong own teeth like me.” (man, 78, tetraplegia, severely frail)."

When assessed in relation to bodily decline, the requirement of a functional mouth in order to eat properly was also linked to dignity:

“There is this lady here, she does not have teeth, and no dentures either. She cannot eat half of what is being served. After every meal the edge of her plate is full of all the stuff she cannot bite. [.] However hopeless my body’s condition is, I wish to eat properly. Otherwise my diet would be down to porridge. That would be horrible.” (woman, 77, severely frail)."

There were however a few, mostly severely, frail participants who tolerated eating difficulties without complaint “*You only spend half an hour a day eating anyway*” (woman, 93, severely frail). Without exception, these were people who had been living with chronic disease for years and who showed in their narratives a high degree of acceptance of their health situation.

A few participants, mostly severely frail men in institutions, said that the functionality of their natural teeth was the principal, if not only, reason for not having them replaced by artificial teeth. This opinion was expressed by people who generally seemed to accept their health decline and some decline of oral function without too much difficulty as an inevitable aspect of old age.

#### Appearance

For most participants, ‘good appearance’ equaled looking “*neat*” and “*well cared for*”. Most people thought that natural teeth looked better than artificial teeth, but in case they would negatively affect their appearance, it was time to have them replaced:

"“I would not like to smile at someone if my teeth would look bad. [..] Although I'd love to keep my own teeth, in that case I would rather have artificial teeth.” (woman, 69, moderately frail)."

The men were clearly less concerned than the women about their appearance and most mentioned that they found oral function much more important than dental looks.

However, a typical response for the male and female severely frail was to consider their declining oral appearance in the perspective of their declining general health:

“I have my teeth the way they are. And yes they do get yellow, and yes they are not straight and neat anymore. But I do not mind. That is because I am not in good shape anymore, I think. I don’t care what it looks like anymore, I’m only concerned with my health now.” (woman, severely frail, 83 year)."

Indeed, the few women who seemed unconcerned about their appearance, seemed also quite accepting of their health decline and the thought of death. A certain degree of decline in oral appearance was accepted by most respondents across all frailty categories:

“If you are 75 and you have a beautiful set of teeth, well that’s a strange sight isn’t it? I think that your face is allowed to show that you are not 20 or 30 anymore, no matter if it is about your teeth or your eyes or your skin.” (woman, 75, slightly frail)."

However, if the decline passed a certain point, many participants saw it as unacceptable, which stresses the relevance of personal appearance even at a later age.

The women of Indonesian heritage strongly emphasized the value of their appearance and thought that they were more critical than Dutch people:

“The people here don’t care about their teeth. They don’t have nice teeth and they don’t brush them. When I moved to Holland, it was striking that the Dutch have such bad teeth. […] Whereas all people from Java…their teeth are beautiful.” (woman, 79, severely frail)."

Natural teeth were strongly related to body-image and several participants agreed that you “*get a different face when you have dentures*” (woman, 79, moderately frail), which was something they tried to avoid. Not only the internally constructed body-image (the way someone sees him/herself) but also the externally constructed body-image (the way someone perceives others see him/her) and the body image of others could be affected by the looks of (natural) teeth:

“Your teeth, they […] help create your facial expression, that way they also add to your identity. I think it plays a role in how people see you, the way you look and the way your mouth looks play a role in that. I recently came across a former acquaintance. He had this crooked mouth and I only saw a few teeth. Well that makes someone look so…. I wanted to say ‘decayed’ but that sounds disrespectful. But old, and uncared for. […]. I would like to keep my own teeth. […] I think people judge me differently when my teeth change, the first impression is different.” (woman, 69, slightly frail)."

The idea that natural teeth contributed to personal identity was shared among several participants across all frailty categories and was reflected in comments like: “*you become a different person with artificial teeth*”. Although most people wanted their teeth to look well cared for, they were only prepared to have major imperfections, like missing or rotten teeth in visible positions, restored. Minor imperfections, like skewed or stained or yellow teeth, did not bother them enough to undertake action, mostly because they set their standards based on what they saw around them in their peer group or what they thought would be normal for their age.

#### Comfort

‘Comfort’ addressed in large part the psychological aspects of impairment, including enjoyment of food and absence of embarrassment, and was related closely to function. Most participants who thought that natural teeth contributed functionally to QoL, also thought that natural teeth contributed to a higher level of comfort through absence of worries about eating, speaking, loose teeth, ill-fitting dentures or dental appearance. Being able to enjoy food and the taste of food were the items that were often mentioned in relation to advantages of having natural teeth:

“If I would not have my own teeth, that would be a big loss. I know people who say: I don’t eat this fruit because I can’t have those little seeds underneath my dentures. […] And like in restaurants, I would hate it if I would have to skip menu’s or dishes because of fear of dentures falling out or food sticking to those fake teeth or whatever […]. It would take away the joy of eating out.” (man slightly frail, 72)."

Social activities were sometimes avoided by people with uncomfortable dentures. Problems with speaking or communication caused by impaired oral health were mentioned only on the context of imagining from observing other denture-wearers.

Absence of pain and irritation was crucial to good QoL for people across all frailty categories. A small number of participants reported that they were experiencing orofacial problems during the interview, mostly from ill-fitting removable dentures.

Few participants felt that maintaining natural teeth compared to dentures could require more time and effort. Almost everyone was bothered by attending a dentist, although only a few participants mentioned fear or particularly bad experiences, and one participant stopped seeing a dentist for fear caused by bad experiences.

Fear of loss of decorum was associated with removable dentures:

“I had this aunt, if she had a meal then she dug up a big white handkerchief from her handbag and once she started eating, then she wrapped her dentures in it. That’s something I hope to never experience myself. How awful, when you’re somewhere without your teeth.” (woman, 72, moderately frail)."

On the contrary, a few participants who were content with their partial removable dentures, explained that if their natural teeth caused problems, they would have to be removed.

#### Adaptation and coping

Participants generally experienced deteriorating teeth as something that inevitably happens with age, and so they reasoned that “*if you can*’*t change the situation*, *you should accept it and cope with it*.” Participants who were very frail who felt that their oral health was poor seemed particularly resigned in this way:

“It is easy for me to accept that my teeth are getting worse. I don't really mind. It is something you can't change anyway. […] Everything gets worse with age” (woman, 85, severely frail)."

Several ‘younger’ or slightly frail participants remarked that they did mind losing their teeth now, but that they expected to accept it with more ease with increasing frailty:

“I am still relatively young now, but when I would be 85 or 90, I expect I would have a different view, depending on my general health. If my health would not further deteriorate, I would still think the same about my mouth, but I expect that I would care less if I would be demented or have other ailments that affect my life and that I cannot control. It really depends on which diseases I would have and how bad they would be.” (man, 69, slightly frail)."

Participants who did not mind losing their teeth, seemed also resigned to a deterioration in their general health. The adaptive strategy used was to anticipate oral health decline through lowering expectations, to compare themselves to others who had lost their teeth, or to judge the importance of teeth in relation to other life and health events:

“Throughout the years, you don’t know if your teeth are still important to you or not.[.] So many things play a role, like with my health in general. I can hardly walk anymore, I had to move to this home, so many things changed. [.] I suppose it made me less concerned about my teeth.” (woman, 84, moderately frail)."

## Discussion

This study revealed how having, caring for and having preserved natural teeth in general improved the quality of life of frail older people through a sense of achievement, pride, a sense of control, intactness, oral function, comfort and appearance. We identified a not previously documented response, especially by severely frail people with chronic pain, that involved clinging to an intact body part (natural teeth) as a means to preserve self-worth, in particular through pride, a sense of control, and a sense of intactness. This is also the first study to indicate how particular frailty aspects (chronic pain and impaired fine motor skills) and the degree of frailty modify the relation between QoL and having natural teeth.

Both quantitative studies [25,26,30,42,43] and qualitative studies [28,29] have identified the positive contribution of natural teeth to QoL, but only MacEntee and his collaborators [28] have addressed this contribution in some detail. Their observations largely correspond with ours, but, as natural teeth were not the focus of their research, they did not provide a comprehensive analytical context and identify specific factors that reflect the value of having natural teeth, or identify the positive effect of natural teeth on self-worth and personal identity, as we did.

### Strengths and limitations of the study

One of the strengths of our study is that its design enabled differentiation between participants with different levels and characteristics of frailty. Hence we were able to compare responses between people of different degrees of frailty and with different frailty characteristics, even though comparisons based on frailty characteristics did not reveal obvious differences other than those related to chronic pain and impaired fine motor skills.

By focusing on natural teeth and its contribution to QoL in 38 lengthy interviews, we were able to cover the subject in more depth than previous studies that focused on oral health in general. Moreover, by explicitly asking what constituted QoL for the respondent before asking about the contribution of natural teeth to QoL, we could explore the value of natural teeth to QoL domains that were deemed important by the participant.

Apart from chronic pain and loss of fine motor skills, loss of cognitive function is probably another strong, frailty-related, influence on oral health related QoL [4], but our interviews were limited to elders who were cognitively alert. Nor did we include edentulous people because we were primarily interested in the value of natural teeth to QoL of frail elders. However, older people without natural teeth could add insight to the value of having natural teeth by comparing their experiences before and after tooth loss, an experience that is generally but not always unpleasant [28,31,33]. We looked at coping and adaptation, which we expected to be the most relevant personal aspects in relation to our study aims, but not at other personal traits like neuroticism, extraversion, and openness, which may [44] or may not [45] influence dental perceptions. Likewise, in our analysis, we did not account for socio-economic status (SES), even though there is evidence that higher SES has a positive influence on OHrQoL [46,47].

The influence of cultural background could not be comprehensively evaluated, since our study included only two people from non-European heritage, which was due to the lack of non-European dentulous elderly who live in assisted living homes or frequent daycare centers in East-Netherlands.

### Meaning of the study: possible explanations and implications for research

The impact of achievement and pride, intactness and sense of control in relation to having natural teeth seemed to be the most obvious for severely frail, institutionalized people. This impact can be understood with help of social comparison theory [48] and a theoretical model from educational psychology: The internal/ external frame of reference model [49]. According to this model, students base their self concepts on two simultaneous sets of comparisons. The internal comparison (or “frame of reference”) includes an individual student’s appraisal of competence in one academic area compared to his or her competence in other academic areas. The external comparison is the student’s appraisal of his or her competence in that academic area relative to the perceived ability of peers, following social comparison theory.

Likewise, our participants, by attributing value to having natural teeth, compared their oral status both externally with their peers and internally to other health areas e.g. their own mental health or motor abilities. For the most severely frail dentulous elderly, both external and internal comparisons are likely to contribute more to a concept of self in a positive way [50], than for slightly frail or non-frail dentulous elderly. The severely frail, especially if they are institutionalized, are more often surrounded by other severely frail people, who are more likely to be toothless than less frail or non-frail elders [51]. Hence, when severely frail dentulous elders compare themselves to their, mostly edentulous, peers (external comparison), they feel more special since they are one of the very few who still have natural teeth.

Making an internal comparison, people value their dental status in comparison to other health areas. Dentulous frail older people realize that their teeth have remained in relatively good condition while other parts of their body have declined. When the decline in other health areas is more severe, the contrast with healthy teeth is even greater, and teeth can contribute even more significantly to self-worth.

In contrast, the experiences of increasing frailty can help prepare people to cope and accept tooth loss, which corresponds with current beliefs about coping resources and declining health [17,52].

This study revealed the contribution of having natural teeth to a positive body-image, not only through dental appearance, but also through intactness and normal functioning, all of which aspects are integrated in the body-image concept as described by Carver [53]. Donnelly [54] indicated how oral impairments could negatively affect the body image of elderly people and consequently decrease self-esteem. She warned that elderly, living in a society where the emphasis is on youth and beauty, may become increasingly concerned about their dental appearance and feel inadequate when they do not have white and straight teeth. Most of our participants, however, did not mind that their teeth were a bit yellow and misaligned. They were more concerned about keeping their own teeth, since artificial teeth made them ‘feel like a different person.’ This association between natural teeth and identity (of which body image is a ‘central aspect’ [55]) at old age, may become more important to OHrQoL as people age and become more frail, than the mere aesthetic aspects of teeth. Most consulted literature indeed supports the idea of decreasing emphasis on physical attractiveness in relation to QoL as people age [56-59], while the experience of bodily decline appears to “urge old people to redefine their identity” [60]. However, further research in the area of oral health is required to test our hypothesis.

The way natural teeth can contribute to a more positive body image and self-worth, cannot be measured by existing OHrQoL instruments. More in general, and to our surprise, body image assessment has not been integrated into the oral health related QoL literature, and has only recently become a topic in health related QoL literature [61-64], despite consistent observations that changes in physical appearance, function, and body integrity are crucial to the experience of health and illness [65]. It may therefore be useful, when researching the OHrQoL of frail elderly, to supplement commonly used OHrQoL instruments like OHIP and GOHAI with questions that target the influence of oral health on body-image and self-worth, e.g. “Do you think that your teeth positively contribute to (a) how others perceive you; (b) how you perceive yourself.”

### Implications for the health sector, health care staff and the dental profession

We found that the severely frail people were less able and less prepared to take good care of their teeth, despite the value they attributed to having natural teeth. There seems to be a turning point where frail people abandon oral care, and our participants indicated that this occurred when they experienced other more disturbing discomforts or pain. At the same time, our results show that even the most severely frail generally wish to keep their natural teeth and benefit from keeping them. Both the health care and public health sector should become aware of the QoL benefits of preservation of natural teeth even for severely frail people. We recommend the dental profession and health care staff to adopt a patient-centered approach through identifying individual oral health needs and wishes of frail dentulous elderly and translating these into a tailor made care plan. In identifying those needs, health care staff needs to be alert to care behavior and the general oral condition. Several of our participants had unclean teeth and simply wanted to be reminded about or help with brushing their teeth or with dental visits, so enhancing their QoL may not require that much effort. However, the required effort needs to be facilitated by the health care and public health sector through allocation of appropriate resources. Only then, the type of requested oral health care, including assistance with daily oral care and arrangement of dental visits, can be better geared to preserve teeth and sustain QoL of frail older people, than is currently the case.

## Conclusions

Participants generally agreed that having, caring for and having preserved natural teeth contributed to their QoL through a sense of achievement, pride, sense of control, intactness, better oral function, more comfort and nicer appearance. The impact of achievement and pride, intactness and sense of control in relation to having natural teeth seemed to be the most obvious for severely frail, institutionalized people.

In the course of increasing frailty, preservation of teeth can help to enhance a positive body image and self-worth, and positively influence QoL.

## Abbreviations

QoL: Quality of life; OHrQoL: Oral health related quality of life; OHIP: Oral Health Impact Profile; GOHAI: Geriatric/General Oral Health Assessment Index.

## Competing interests

The authors declare that they have no competing interests.

## Authors’ contributions

DN designed the study, carried out the interviews, transcribed and analyzed the data and wrote the paper. KM carried out the interviews, transcribed and analyzed the data and contributed to the paper. WS analyzed the data and contributed to the paper. All authors have read and approved the final version of the manuscript.

## Pre-publication history

The pre-publication history for this paper can be accessed here:

http://www.biomedcentral.com/1471-2458/12/839/prepub
